# Overlap Intensity: An ImageJ Macro for Analyzing the HIV-1 In Situ Uncoating Assay

**DOI:** 10.3390/v13081604

**Published:** 2021-08-13

**Authors:** Zachary Ingram, Hannah Matheney, Emma Wise, Courtney Weatherford, Amy E. Hulme

**Affiliations:** Department of Biomedical Sciences, Missouri State University, Springfield, MO 65987, USA; Ingram13@live.missouristate.edu (Z.I.); Hannah023@live.missouristate.edu (H.M.); Emma230@live.missouristate.edu (E.W.); Courtney145@live.missouristate.edu (C.W.)

**Keywords:** HIV-1, capsid, uncoating, ImageJ, image quantification, fluorescent microscopy, fluorescent overlap

## Abstract

Capsid uncoating is at the crossroads of early steps in HIV-1 replication. In recent years, the development of novel assays has expanded how HIV-1 uncoating can be studied. In the in situ uncoating assay, dual fluorescently labelled virus allows for the identification of fused viral cores. Antibody staining then detects the amount of capsid associated with each viral core at different times post-infection. Following fixed cell imaging, manual counting can be used to assess the fusion state and capsid signal for each viral core, but this method can introduce bias with increased time of analysis. To address these limitations, we developed the Overlap Intensity macro in ImageJ. This macro automates the detection of viral cores and quantification of overlapping fusion and capsid signals. We demonstrated the high accuracy of the macro by comparing core detection to manual methods. Analysis of an in situ uncoating assay further verified the macro by detecting progressive uncoating as expected. Therefore, this macro improves the accessibility of the in situ uncoating assay by replacing time-consuming manual methods or the need for expensive data analysis software. Beyond the described assay, the Overlap Intensity macro includes adjustable settings for use in other methods requiring quantification of overlapping fluorescent signals.

## 1. Introduction

The viral capsid is critical for multiple early steps of HIV-1 replication. The conical capsid is made of ~1500 monomers of the viral capsid (CA) protein which surrounds the genomic RNAs and other associated proteins to form the viral core [1,2]. The capsid is implicated in the protection of the reverse transcription complex (RTC), microtubule assisted transport, and nucleoporin interactions [3,4,5,6]. The disassembly or remodeling of the capsid, a step termed uncoating, is a requirement for replication with hyperstable and unstable capsid mutants associated with decreased infectivity [3,7]. Furthermore, the process of uncoating also has an interplay with reverse transcription and nuclear import of the viral genome [8,9,10,11]. The ability to study the capsid and uncoating has improved due to recent advancements in the field.

There are multiple assays available to study uncoating including capsid core stability assays, the fate of the capsid assay, the CsA washout assay, and fluorescence microscopy-based uncoating assays [1,3,12,13,14,15,16,17,18,19,20,21,22,23,24,25]. Each of these assays has different strengths and weaknesses, so often a combination of assays is used to study the factors that influence uncoating. Several uncoating assays have been developed utilizing fluorescence microscopy with capsid detection by antibody staining or through the use of fluorescent markers [8,15,16,17,19,20,21,22]. Collectively, fluorescence microscopy techniques allow the process of uncoating to be studied in infected cells with direct visualization of the capsid and the process of uncoating relative to other cellular structures or proteins. However, these assays are dependent of optimal fluorescent labelling and staining of virus. Some of these fluorescent microscopy assays may also focus on a bulk population of virions, some of which may be uninfectious.

Most recently, fluorescent microscopy-based uncoating assays have been developed that utilize live cell imaging to track the progression of early steps of replication (uncoating, reverse transcription, cytoplasmic transit, nuclear import) in single virions and, in some assays, infection of the target cell [15,16,17,22,23,24,25]. Despite the importance of these experiments, the equipment needed to maintain live cell imaging can be limiting to research institutions. A more accessible alternative to live microscopy-based experiments is the in situ uncoating assay, which relies on fixed cell imaging over a time course [8,19]. The in situ uncoating assay is a confocal microscopy-based experiment where dual-labelled HIV-1 is used to infect cells. The dual-labelled virus includes either GFP-tagged Vpr or integrase (IN) viral proteins [18,26,27]. The GFP-tagged proteins associate with the viral core and act as a marker for its location in the cytoplasm and nucleus of the cell. In addition, the virus producer cells are transfected with a S15-dTomato plasmid. S15-dTomato includes the N-terminal segment of c-Src which embeds into the cell membrane [27,28]. As progeny virions bud from producer cells, the tagged host membrane is incorporated into the HIV viral membrane. Inclusion of the S15-dTomato into the viral membrane allows fusion to be tracked with the loss of the dTomato signal. In the in situ uncoating assay, cells are infected with this dual-labelled virus and then fixed over a time course. Fixed cells undergo antibody staining for CA with a Cy5-conjugated secondary antibody. The Cy5 signal allows the extent of uncoating to be quantified by either the percentage of Cy5-positive virus or the mean maximum Cy5 intensity at different times post infection [19,29]. Since its development, the in situ uncoating assay has been used to examine general uncoating kinetics, the effects of capsid mutations on uncoating, and the impact of reverse transcription on uncoating [7,8,11,19,30]. More recently, the in situ uncoating assay has been instrumental in examining the role of multiple cellular factors (Dynein, Kif5B, FEZ1, BICD2, Nup358, Dia1, Dia2, EB1, CLIP170) involved with cellular trafficking and nuclear import on uncoating [14,29,31,32,33,34]. These findings have been further supported by alternate uncoating assays or by characterizing related replication steps. Therefore, the in situ uncoating assay is a valuable tool for revealing different aspects of the early steps of HIV replication.

Following the imaging of fixed cell samples in the in situ uncoating assay, images must be analyzed to determine the extent of uncoating. First the GFP-positive viral particles must be sorted into fused (dTomato-negative) and unfused (dTomato-positive) categories. GFP-positive viral particles that have fused are then measured for Cy5 intensity as the primary readout of capsid. Typically, the majority of imaged puncta represent unfused cores resulting in a lower population of fused virus for analysis [8,14,29]. To quantify a sufficient number of fused viral cores, multiple images are necessary. Increasing the number of images per sample increases the total viral core count but extends the time of data analysis. Several approaches can be used for data analysis. One option is to manually count GFP signals before sorting each virus into fused, unfused, coated, and uncoated groups. This manual analysis is carried out through the placement of regions of interest (ROIs) around GFP signals. Total ROIs are counted, followed by visual inspection for dTomato and Cy5 signal that overlaps with the ROI. While the manual method can be utilized with a variety of image processing software applications, the analysis proves to be laborious. Potential bias may also be introduced as a dim GFP signal may be undercounted or vary between individual counters. In instances where the signal is low or a confocal system lacks sensitivity, virus detection can be inaccurate and imprecise under visual inspection. An alternative to manual counting is the use of semi-automatic methods. Semi-automatic analysis requires an established protocol or macro within an image processing software, but user bias is removed with automatic detection of virus labelling. Rapid detection and measurement of signal overlap further eliminate the downsides found in the manual method. Currently no freely available software is designed for detection and quantification methods specific to the in situ uncoating assay. Software that has been used in previous research, such as Imaris and MetaMorph Imaging Software, are capable of analysis but are limited by the cost for software access [14,29].

ImageJ is a free open source alternative to cost restrictive imaging software [35]. ImageJ includes a flexible toolset for image processing with plugins available to expand analysis options. Within ImageJ is a set of adaptable tools, including scripting capabilities, to develop macros and plugins. Building on the ImageJ framework, Fiji Is Just ImageJ (FIJI) represents a packaged version of ImageJ with the inclusion of various plugins and tools [36]. The colocalization analysis found in FIJI or ImageJ cannot be used for the in situ uncoating assay. Colocalization measures the degree of overlap between two signals, which is often expressed as the Pearson’s correlation coefficient or Manders coefficient. The output of these analyses does not include a fluorescence intensity for each fluorophore in an ROI. When using the current capabilities of ImageJ for the in situ uncoating assay, GFP punctate can be manually counted as ROIs followed by the measurement of dTomato and Cy5 intensity in each ROI. However, the large number of particles counted contributes extensive time spent processing images during analysis, as previously described.

In this work, we have developed the semi-automated Overlap Intensity macro for ImageJ to automatically count GFP signal and then measure dTomato and Cy5 intensity in each ROI. We validated our macro’s ability to detect GFP signal above background against a manual counting method. Additionally, we used this macro to analyze data from the in situ uncoating assay. In developing this macro, we hope to expand the accessibility of the in situ uncoating assay. Importantly, we have designed the Overlap Intensity macro with adjustable settings to allow for use beyond the described uncoating experiment. The Overlap Intensity macro is available for download at https://github.com/HulmeLab/Overlap-Intensity-Macro (accessed on 11 August 2021).

## 2. Materials and Methods

### 2.1. Cell Lines and Pharmaceuticals

The 293T HEK cell line was received from the Hope lab at Northwestern University [8]. 293T cells were cultured in Dulbecco’s Modification of Eagle’s Medium (DMEM, Corning), 1% Penicillin/Streptomycin/L-Glutamine (PSG, Corning), and 10% Fetal Bovine Serum (FBS, R&D Systems, Minneapolis, MN, USA). The human microglial cell line CHME3 was received from the Naghavi lab at Northwestern University [37]. CHME3 cells were maintained in DMEM with 1% PSG, 5% FBS, and 0.91 mM Sodium Pyruvate (Corning). All cell lines were maintained at 37 °C with 5% CO_2_. Baflomycin A (BafA, Sigma, St. Louis, MO, USA) was stored at −20 °C and used at a working concentration of 0.02 uM. DEAE Dextran (Sigma) was used at a concentration of 10 ng/µL and was stored at 4 °C. Polyethyleneimine (PEI) was aliquoted in sterile ddH_2_O and stored at 4 °C.

### 2.2. Virus Production and Characterization

Dual-labelled VSV g pseudotyped HIV-1 was produced through PEI transfection of 293T HEK cells with four plasmids as previously described: CMV-VSV-G, HIV-GFP, GFP-Vpr, and S15-dTomato [27]. The HIV-GFP proviral plasmid has mutations present in the Env gene to render it replication defective (ΔEnv, [38]). In this plasmid, GFP is cloned into the Nef position and functions as a GFP reporter for infection of the cell. For the imaging experiments described here, the GFP reporter does not impact GFP labelling when making dual-labelled virus. Virus was harvested by passage of transfected cell media through a 0.45 um syringe filter (Millex-HV) before storing virus aliquots at −80 °C. Virus was characterized on glass to confirm GFP co-labelling with dTomato and CA staining percentage of at least 80% and 50%, respectively. Sterile glass coverslips were transferred to a 24-well plate before a 15 min treatment with Poly-L-Lysine (Sigma). Dual-labelled virus was added to the subbed glass coverslips at 3 concentrations (undiluted, 1/10 dilution, and 1/20 dilution) to determine the concentration used for future experiments. Plated virus was then centrifuged for 1 h at 1200× *g*, 16 °C. After centrifugation, all coverslips were fixed with 4% paraformaldehyde for 15 min. Coverslips were then washed with PBS, permeabilized with 0.05% Triton X in PBS for 15 min, and blocked with 10% FBS solution for 45 min. Primary antibody staining with the human Anti-HIV-1 p24 monoclonal antibody (241-D) was carried out overnight at 4 °C [39,40,41]. Secondary antibody stain was carried out with an anti-human 647 antibody (Jackson ImmunoResearch) at room temperature for 1 h. Coverslips were mounted onto glass slides using ProLong™ Glass Antifade Mountant and imaged using confocal microscopy.

### 2.3. Validation Assays

For virus on glass validation assays, sterile glass coverslips were transferred to a 24-well plate before a 15 min treatment with Poly-L-Lysine (Sigma). For virus on cell validation assays, glass coverslips stored in 70% ethanol were transferred into 24-well plates and treated for 30 min with EmbryoMax 0.1% Gelatin (MilliporeSigma) at room temperature. CHME3 cells were plated at 120,000 cells per well and incubated overnight. Dual-labelled virus was applied to the coverslips at 1/10th, 1/20th, 1/30th, and 1/40th dilutions in media. Plates were then centrifuged for 1 h at 1200× *g*, 16 °C. After centrifugation, all coverslips were immediately fixed with 4% paraformaldehyde for 15 min. Antibody staining and coverslip mounting were carried out as described in the Virus Production and Characterization section. Coverslips were imaged using confocal microscopy.

### 2.4. In Situ Uncoating Assay

Glass coverslips stored in 70% ethanol were transferred into 24-well plates and treated for 30 min with EmbryoMax 0.1% Gelatin (MilliporeSigma) at room temperature. CHME3 cells were plated at 120,000 cells per well and incubated overnight. Infection was carried out with a 1/10th dilution of dual-labelled virus with 1X DEAE Dextran. A cell only condition was included with mock infection to determine cell background. As a fusion control, one coverslip underwent four hours of infection with constant BafA treatment. Cells underwent spinoculation for 1 h at 1200× *g*, 16 °C. Following spinoculation, the infection media was aspirated with the addition of 37 °C culture media initiating the infection time course. At each time point, coverslips were fixed with 4% paraformaldehyde for 15 min. Antibody staining and coverslip mounting were carried out as described in the Virus Production and Characterization section. An antibody control was included by staining one infected coverslip with only the secondary antibody. Coverslips were imaged using confocal microscopy.

### 2.5. Confocal Microscopy and Image Processing

Fixed coverslips on glass slides were imaged with a Leica SP8 DMI8 confocal microscope using the Leica Application Suite X (LASX) software (Leica Microsystems, Buffalo Grove, IL, USA). All images were acquired with a pinhole size of 1.00 AU. GFP, dTomato, and Cy5 signal were sequentially captured with 488 nm, 532 nm, and 635 nm wavelength lasers. For each experimental condition, 10 images were acquired as a Z-stack. For each image, the Z-stack was maximum projected into a 2D image. Using the LASX software, images were then thresholded to remove background in the GFP, dTomato, and Cy5 channels. The background level thresholds were determined for GFP and dTomato according to the stained cell only control. Cy5 background threshold was set based on the secondary antibody only negative control. After thresholding for background, images were exported as TIFFs for analysis in ImageJ.

## 3. Results

### 3.1. Establishment of the Overlap Intensity Macro

**Figure 1:**
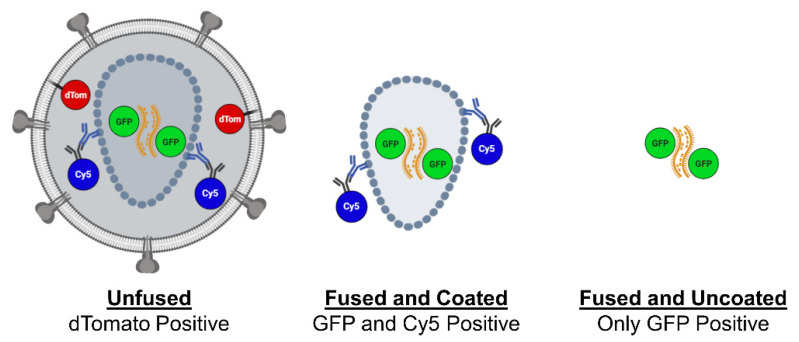
Schematic of dual-labelled HIV-1. Dual-labelled virus used in the in situ uncoating assay can include various combinations of fluorophore signal. Pre-fusion virus is expected to appear dTomato-positive. Post-fusion the dTomato signal is shed, while GFP and Cy5 signal remains. Loss of capsid is identified based on GFP-positive but Cy5-negative punctate. Image created with Biorender.com.

**Figure 2:**
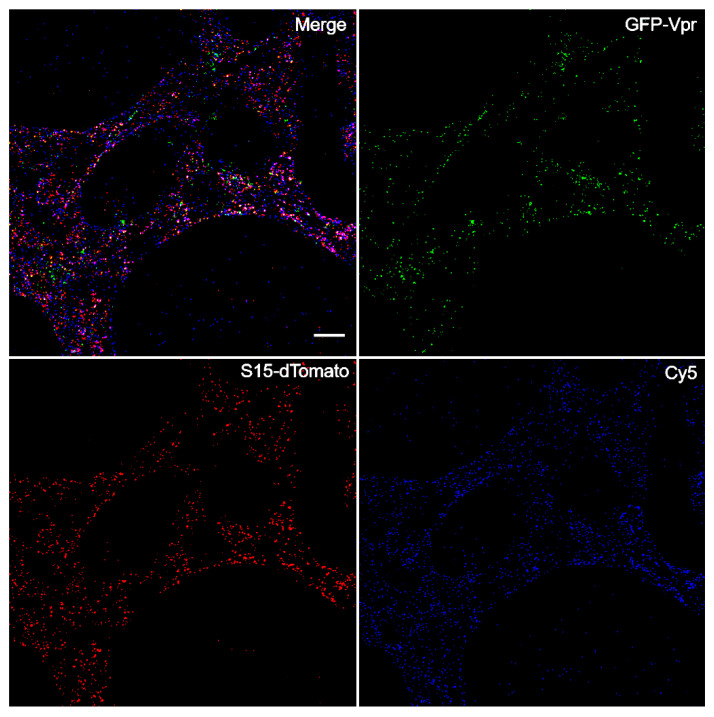
Representative image of the in situ uncoating assay. CHME3 cells were infected with dual-labelled VSV g HIV-GFP before fixing and imaging. GFP signal is represented in the green channel. The dTomato fusion marker is shown in red. Antibody capsid stain is visible in the blue channel representing a pseudocolored Cy5 signal. The white scale bar in the Merge panel is 10 µm. Image brightness has been enhanced for signal visibility.

Upon completing an in situ uncoating assay experiment, the data analysis requires categorizing and sorting individual viral cores based on labelling. Various combinations of the three fluorescent labels allow for each virus to be identified as either unfused, fused with detectable capsid signal, or fused with no detectable capsid signal (Figure 1). Virus is identified based on GFP-positive punctate marked by GFP-Vpr. The presence of dTomato signal indicates unfused virus that remains associated with the viral envelope. Viral cores that have fused are identified by absence of the dTomato marker. Virus can then be assessed for CA present based on the Cy5 signal (Figure 2). The sorting of viral cores into each category precedes data analysis. To properly assess uncoating, only virus that has fused is included in the final analysis. While counting and sorting can be completed manually, the process proves to be time consuming. Furthermore, variability between counters and images can introduce errors and bias. To expedite the assessment of dual-labelled virus, the Overlap Intensity macro was developed within ImageJ. The goal in designing this macro was to uniformly identify viral punctate above cell background while assessing the intensity of the fluorescent signal across multiple channels. In this way, the Overlap Intensity macro provides a basic readout of overlapping signal intensity.

The Overlap Intensity macro functions through automating GFP detection and quantification of multiple fluorophores. Data analysis can include all parameters found within ImageJ’s suite of measurements that pertain to ROIs. Prior to use, the macro must first be installed by copying the Overlap_Intensity.ijm to the ImageJ Plugin folder. The macro will then be found under the Plugins tab. Running the macro will open the settings menu. Here, the channel for particle analysis can be selected, size parameters set, channels for measurement selected, and an optional processing check can be toggled. Importantly, the global scale of the images used must be set in ImageJ for proper particle analysis. The macro is compatible with RGB and composite images. Images can be analyzed individually, or multiple images can be analyzed at one time using the batch process option.

**Figure 3:**
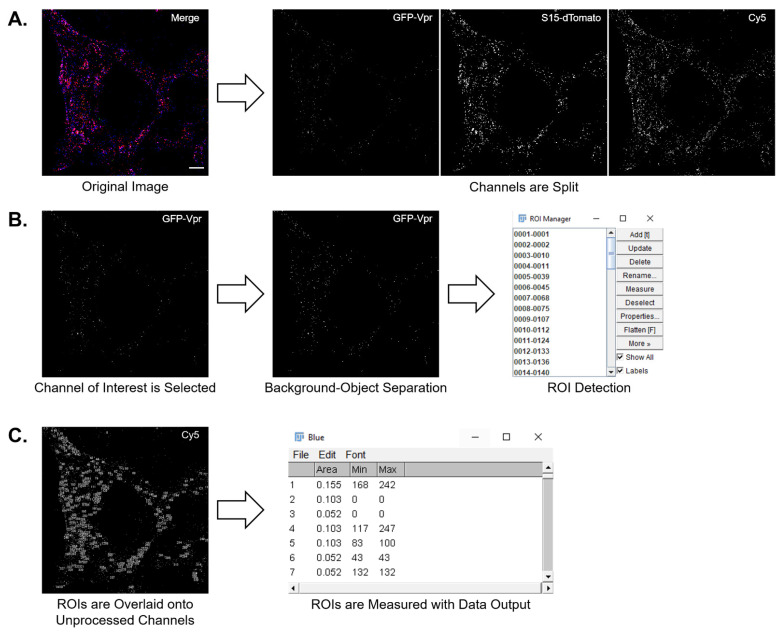
Macro workflow. (**A**) A background adjusted multicolor image is split into individual channels, each representing a single fluorescent signal. In this example, green, red, and blue correspond to GFP, dTomato, and Cy5, respectively. The white scale bar in the Merge panel is 10 µm. Image brightness has been enhanced for signal visibility. (**B**) The channel for particle location is selected and undergoes auto-thresholding. Remaining objects are identified and stored as ROIs. (**C**) The stored ROIs are overlaid onto the unadjusted blue and red channels before measuring each ROI individually. Data can then be exported to a separate software for further analysis.

In the macro workflow, fluorophores are first separated by splitting an image into individual channels that represent each signal (Figure 3A). We have provided the option to select all available channels in ImageJ, which includes green, red, blue, gray, cyan, magenta, and yellow. Next, a primary channel is selected to identify ROIs and undergo auto-thresholding in the ROI channel using a thresholding algorithm. The macro allows thresholding settings to be changed to any of the 16 methods included in ImageJ. To ensure that background has been sufficiently removed during thresholding, we included a process check option. By selecting the check option in the settings menu, the macro will pause post-thresholding. This step allows thresholded images to be viewed before continuing with the analysis. For the in situ uncoating assay, we selected the green channel for GFP-Vpr to identify ROIs (Figure 3B). The green channel then underwent auto-thresholding to separate objects from background. In the work shown, a max entropy auto-threshold was applied to all images. Successful separation of objects from background is dependent on the signal difference between the background and punctate. As a result, the use of a max entropy threshold may not be appropriate for all types of assays. The processed image then undergoes particle analysis with adjustable size parameters. Restrictions can be placed on detection limits by setting the maximum and minimum size. Additionally, object circularity can be used to gate objects in a range of 0.00 to 1.00. For the in situ uncoating assay, particle analysis was set to identify objects between 0.00 and 2.00 μm^2^ to exclude any remaining cell background being identified as an ROI. With near pixel size punctate for all viral cores, the object circularity was set to 0.00 to 1.00. The completion of particle analysis results in saving each particle as an ROI (Figure 3B). By logging each ROI, the location, shape, and size of a particle is available. This maintains the area in which GFP was detected, allowing for overlapping signals in other channels to be measured. With ROIs recorded, the macro then overlays each ROI onto the channels chosen for measurement (Figure 3C). In the in situ uncoating assay, dTomato signal is identified in the red channel as the fusion marker. A pseudocolored Cy5 shown in blue represents capsid presence. Each parameter selected is then measured and presented within a results table (Figure 3C). Results for each channel are listed as the color name to differentiate between a multichannel measurement. In the context of the in situ uncoating assay, the maximum signal intensity associated with the ROI is quantified. The maximum signal intensity is preferred over total or average intensity as this measure is independent of the size of the ROI. Data for each ROI were then exported to Microsoft Excel for calculations based on dTomato and Cy5 intensity.

### 3.2. Validation

**Figure 4:**
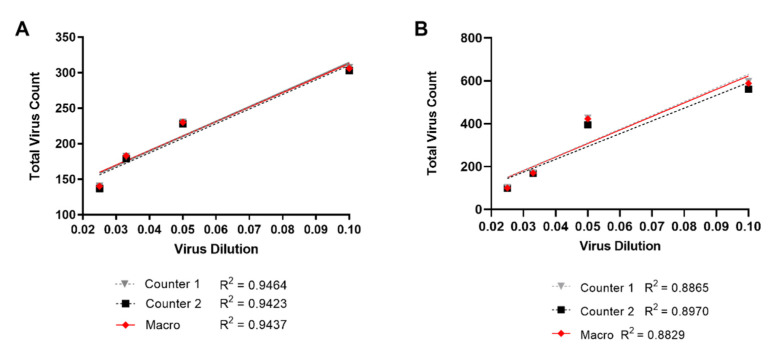
Virus identification comparison between manual counting and the Overlap Intensity macro. Dual-labelled virus signal at 1/10th, 1/20th, 1/30th, and 1/40th dilutions was adhered to glass (**A**) or added to CHME3 cells plated on glass coverslips (**B**) and imaged for GFP. Ten images were taken per dilution. Two independent counters manually identified ROIs for all dilutions. The Overlap Intensity macro was used to assess each dilution. A line-of-best fit was generated for the manual counts and compared to GFP counts from the automatic method. Representative experiments are shown.

To ensure that the macro properly identifies virus based on GFP signal, we compared our macro against two manual counters using a dilution series of virus. Dual-labelled virus was adhered onto glass coverslips at 1/10th, 1/20th, 1/30th, and 1/40th dilutions in media. All coverslips underwent the same infection and CA antibody staining conditions used in the in situ uncoating assay. Ten images were captured per dilution to ensure a sufficient amount of virus for counting comparison. GFP punctate was then manually counted by two independent counters. Images were auto-thresholded before ROIs were manually drawn over each image and counted. The Overlap Intensity macro was then used to quantify total number of GFP signal and compared against the manual counts. Total counts per dilution were plotted with a line of best fit for each method (Figure 4A). We found manual and automatic counting methods were in high agreement with a near identical count at all dilutions. Furthermore, each trendline closely overlapped with a near 1.0 R^2^ value indicating the expected correlation between virus dilution and count. Virus on glass experiments typically have a low and uniform level of background, which would make identifying virus easier. However, in the in situ uncoating assay, virus must be identified on a background of adherent cells. Therefore, we repeated the same validation assay but applied the dilution series of virus to CHME3 cells adhered to glass coverslips. Similar to the previous validation assay, there was high agreement in virus count at each dilution between the manual counters and the Overlap Intensity macro (Figure 4B).

### 3.3. Application

**Figure 5:**
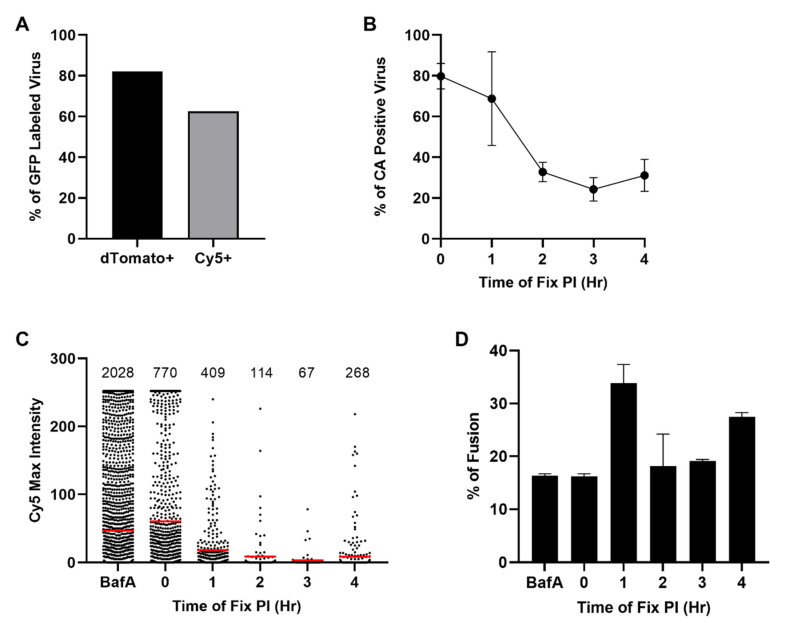
Analysis of in situ uncoating assay data. The validation of virus labelling and in situ uncoating assay was carried out and analyzed using the Overlap Intensity macro. (**A**) Dual-labelled virus was adhered to glass and imaged. GFP-positive cores were identified by the macro before measuring each ROI for dTomato and Cy5 signal. The percentage of dTomato and Cy5-positive cores was calculated based on the total number of GFP-positive cores. (**B**) Total percentage of Cy5-positive viral punctate was determined for 0, 1, 2, 3, and 4 h post-infection. Percentage was determined based on the total number of fused GFP punctate with detectable Cy5 signal compared to total number of fused GFP punctate. Shown is an average of two independent experiments with error bars denoting standard error. (**C**) All fused GFP-positive cores were quantified for Cy5 signal and plotted as a scatterplot. Cy5 signal was assessed and plotted for all fused and unfused cores for the BafA control and 0 h time point. The red bar represents the mean of the maximum Cy5 intensity associated with each viral core. Viral core counts are represented above each column. Data from two independent experiments are shown. (**D**) The percentage of fused cores was calculated based on the number of dTomato-negative cores divided by the total viral core count. Shown is an average of two independent experiments with error bars denoting standard error.

The in situ uncoating assay is used to assess the progression of HIV-1 uncoating. A requirement within the assay is the dual-labelling of HIV-1 with S15-dTomato and GFP-Vpr. Suboptimal fluorescent labelling or CA staining can interfere with results from the assay, so virus is first validated for fluorescent labelling. To limit background levels of unfused virus that could obscure results, each stock of virus must have 80% or higher of GFP-positive cores that are co-labelled with S15-dTomato. Following antibody staining for CA, all GFP-positive cores must show 50% or higher Cy5 signal. Viral stocks are deemed unusable if either parameter is not met. To validate our virus stock, we adhered virus directly onto glass. Samples then underwent spinoculation and antibody staining to mimic the in situ uncoating assay. Images were then passed through the Overlap Intensity macro to determine labelling percentages. GFP-positive virus was found to have 82.1% dTomato labelling with 62.6% positive for Cy5 (Figure 5A). Therefore, this virus stock demonstrated sufficient labelling for use in the in situ uncoating assay. All experiments shown in this work utilized this verified stock of virus.

In developing the Overlap Intensity macro, our goal was to reduce the time of analysis in the in situ uncoating assay while maintaining accurate quantification. To ensure that the macro properly detects GFP punctate and can measure associated CA signal, in situ uncoating assays were carried out [8,19]. CHME3 cells were infected with dual-labelled HIV-1 and fixed at 0, 1, 2, 3, and 4 h post-infection. Mock infected cells underwent an identical procedure allowing cell background levels for GFP and dTomato to be thresholded post-imaging. A secondary antibody only control was included allowing Cy5 background to be detected while ensuring specific antibody staining. To assess dTomato labelling, one coverslip of cells was infected under constant Bafilomycin A (BafA) treatment before fixing the cells at 4 h post-infection. BafA is a V-ATPase inhibitor that prevents endosomal fusion of VSV g HIV-GFP [42,43]. The BafA-infected cells should maintain a high percentage of dTomato-positive virus due to inhibited fusion. Similarly, the 0 h condition should indicate a low level of fusion due to immediate fixation. The BafA and 0 h conditions are also expected to represent the maximum capsid signal with uncoating unlikely prior to fusion. Following imaging of the fixed coverslips, images were analyzed with the Overlap Intensity macro. GFP signal was identified as ROIs before measuring red and blue channels for maximum dTomato and Cy5 signal. The GFP signal was size restricted to 0.0 to 2.0 μm^2^ in size to exclude any remaining cell background.

Data from the in situ uncoating assay can be plotted in several ways. When plotting these data as the percent of CA-positive virus, all fused cores with detectable Cy5 signal are categorized as coated (Figure 5B; [8,11,19]). The total fused count is then used to determine what percentage of virus has detectable capsid at each time point. The BafA-treated cells and 0 h time point are not expected to have substantial fusion and therefore were analyzed for all cores regardless of fusion state. The percentage of coated virus was found to decrease with time (Figure 5B). The 0 and 1 h timepoints represented the highest coated percentages at 79.8% and 68.8% (Table 1). Beyond 1 h post-infection, the percentage of coated virus decreased at 2 h post-infection before leveling off for the remainder of the time course. A slight increase in coated virus was detected at 4 h post-infection at 31.1%. The loss of coated particles over time suggests progressive uncoating as expected from previous studies [8,11,19].

Alternatively, the maximum Cy5 signal from each virus can be plotted on a column scatter plot with a calculated mean (Figure 5C; [14,29]). By accounting for the Cy5 intensity of each viral core, sensitivity to changes in capsid association are improved. While the percentage of CA-positive virus provides insight, the use of mean maximum intensity can identify subtler changes in uncoating. The BafA and 0 h time point were found to have the highest Cy5 intensities at 46.9 and 60.5, respectively (Figure 5C; Table 1). As infection progressed, the average Cy5 intensity decreased from 0 to 3 h post-infection (Figure 5C; Table 1). The decrease in mean maximum intensity suggests that viral cores are uncoating over time. In addition, the total number of fused cores detected during imaging decreased with time, which has been previously observed (Figure 5C; Table 1; [8,32]).

Finally, to ensure that variable viral fusion at each time point does not bias these data, the percentage of viral fusion was calculated as the number of dTomato-negative cores divided by the total number of GFP -positive signals (Figure 5D; Table 1). For the BafA and 0 h time point conditions, the extent of viral fusion was 16.3%. We anticipated viral fusion to be less than 20% for both conditions, which was observed. For the remaining time points, the fusion percentages spanned from 18.1% to 33.9%, which is consistent with previous studies (Figure 5D; Table 1; [14,29]).

## 4. Discussion

The in situ uncoating assay is a well-established assay and has been extensively used to study HIV-1 uncoating. Unlike other fluorescence microscopy-based uncoating assays, this assay does not require live cell imaging capabilities. The fixed cell approach of the assay maintains a high amount of virus for quantification, providing a readout of capsid state. However, without semi-automated analysis, throughput remains low. Proper quantification of data first requires identification of ROIs followed by the measurement of overlapping signal intensities across multiple channels. While previous publications have utilized semi-automated approaches, these protocols are not freely available or rely on commercial software [14,29]. Importantly, this analysis is distinct from measuring colocalization and requires a specific workflow not found in imaging software. Therefore, we have developed the Overlap Intensity macro for analysis of the in situ uncoating assay for ImageJ.

During quantification, the primary factor that contributes to the time of analysis is the detection of each fluorophore above background fluorescence. Manual sorting methods prove time consuming and are further complicated by weak fluorophore signal. In instances where dTomato or Cy5 signal is faint, viral cores may be incorrectly categorized as fused or uncoated by manual inspection. GFP punctate may also be incorrectly excluded due to low signal intensities. To remove the bias introduced by visual sorting, the Overlap Intensity macro automates the detection of ROIs and signal intensities. We were able to demonstrate the high accuracy of the macro in GFP detection by comparing ROI counts to a manual approach, both when virus was adhered to glass and bound to cells (Figure 4). There was more variation in the validation assay conducted on cells, which can be seen in the wider spread of points between the manual counters and the macro and the lower R^2^ values (Figure 4B), compared to the assay on glass (Figure 4A). This result is likely due to the increased and non-uniform background fluorescence associated with cells. At all virus dilutions, the semi-automated method for GFP detection was highly similar to the manual counts, indicating comparable accuracy, but by automating the detection process, we have drastically improved time of analysis.

Next, we utilized the macro to successfully assess the in situ uncoating assay. Over time, viral cores will uncoat, resulting in a decrease or loss of Cy5 signal. In agreement with previous work, we found a progressive loss of HIV-1 capsid under two quantifications (Figure 5B,C; Table 1; [8,11,14,19,29]). When quantifying the percent of CA-positive virus, a small amount of uncoating was found between the 0 and 1 h timepoints. After 1 h post-infection, virus rapidly uncoated to less than 40% for the remainder of the time course (Figure 5B). Analyzing the same data by maximum Cy5 intensity indicated a similar loss of CA with time (Figure 5C). However, the mean maximum Cy5 intensity decreased more substantially from 0 to 1 h post-infection. This loss of Cy5 signal indicates that uncoating was detectable shortly after viral fusion. The early detection of uncoating shown through Cy5 signal is likely a result of improved sensitivity. By categorizing virus as simply CA-positive or -negative, differences in CA amounts are lost. In recent years, quantification of these subtle changes has become increasingly important given studies showing evidence of biphasic uncoating, uncoating at the nuclear pore, and nuclear uncoating [6,16,20,23,25,44]. If the imaging system used is appropriately sensitive, the mean maximum intensity provides a better readout of capsid state. In addition, comparing the maximum intensity between time points within an experiment minimizes the effect of suboptimal capsid staining. For this reason, recent studies using the in situ uncoating assay quantify and compare the maximum intensity over time or among different conditions. For example, this method has been used to demonstrate the impact of several cellular proteins involved in cellular trafficking and nuclear import on uncoating [14,29,31,32,33,34]. Despite differences between both methods of analysis, these data demonstrate the Overlap Intensity macro’s ability to detect uncoating. The CA antibody 241-D was used for these experiments, which has been previously used in live and fixed cell imaging experiments [24]. While it is possible that the accessibility of the epitope recognized by this antibody may vary over time, these results are consistent with previous in situ uncoating assays using different antibodies to detect CA [8,11,14,19,29,31].

Beyond the loss of Cy5 signal, various controls must be considered. During early replication, viral fusion precedes uncoating. The in situ uncoating assay typically utilizes VSV g pseudotyped virus so that viral fusion can be synchronized by spinoculation and temperature shift. VSV g-mediated viral fusion occurs by endocytosis, which is different from the canonical entry pathway mediated by HIV-1 Env at the cell membrane. The kinetics of viral fusion from these two methods have been shown to be different, with Env-mediated viral fusion having an increased half-life [8]. However, the method of viral fusion did not impact uncoating kinetics once the different kinetics of viral fusion were taken into account [8]. In the in situ uncoating assay, viral fusion is measured by the loss of dTomato signal and only fused virions are assessed for uncoating. To limit the number of unlabelled virions that could obscure results, dual-labelled virus stocks must first be verified for dTomato labelling of 80% or more before use. Labelling verification includes the detergent-based permeabilization step that reduces the native dTomato labelling during antibody staining. The virus stock used in this work was verified for proper labelling prior to use (Figure 5A). However, due to the importance of the dTomato label, reassessment of the BafA and 0 h condition is necessary within each experiment. The loss of dTomato signal in less than 20% of detectable cores therefore confirms proper labelling and further strengthens data. Differences in the percentage of fused virus could bias the uncoating data by increasing or decreasing the number of fused cores. Therefore, fusion was tracked by calculating the percent of dTomato-negative cores relative to the total count. While incomplete dTomato labelling and low signal above background contribute to some variation, fusion should remain consistent. We were able to show an acceptable range for percentage of fused virus. The lowest fusion rate was found to be 16.3% with peak fusion at 33.9% (Figure 5D; Table 1). The range of fusion over time was 17.6% with an average of 21.9% fused cores. Previous work has shown fusion with percentages ranging less than 20% [11,14,19,45]. The low variation of fusion suggests that these differences do not substantially impact our analysis of uncoating. Within these data, we also observed that less than 20% of total cores were fused for the 0 h post-infection and BafA treatment conditions (Figure 5D; Table 1).

Previous use of the in situ uncoating assay has reported a progressive loss of fused viral cores, distinguished as fewer GFP-positive cores over time [8,32]. Here, we were also able to observe a decrease in the number of fused viral cores (Figure 5C; Table 1). This loss may be explained by the dissociation of GFP-Vpr from the viral core during viral replication. Vpr is shed from coated and uncoated RTCs following fusion, which may further reduce GFP punctate to background levels [46]. Some studies have found that Vpr assists in the nuclear import of the pre-integration complex (PIC) and can localize to the nucleus [47,48,49]. Once nuclear, Vpr may dissociate from the PIC leading to a decreased number of GFP labelled viral cores at later time points. In this study, we were unable to differentiate between cytoplasmic and nuclear GFP labelled viral cores due to the absence of a nuclear envelope marker. Another explanation for the loss of GFP-positive viral cores may be proteasomal degradation of exposed RTCs. Prior to uncoating, the capsid protects the internal RTC from detection and degradation [4,6,50,51]. Uncoated cores are then susceptible to host restriction factors or cell degradation pathways, resulting in the decreased number of fused viral cores at later time points. An alternative to GFP-Vpr is GFP-tagged integrase (IN-GFP), which remains with the PIC following nuclear import [6,26]. However, loss of GFP signal can occur from proteasomal degradation of IN-GFP and the integration of the viral genome [6,52]. Despite the cause of decreasing GFP-Vpr, the Overlap Intensity macro was able to detect this previously reported trend.

While we have shown our macro to be accurate with improved time of analysis, any automated process has caveats. The macro workflow by default utilizes a max entropy auto-threshold to segment images. The use of a max entropy threshold may not properly separate objects from the background for all images. Depending on the background and object intensity, artifacts may be included or objects lost. To accommodate other uses, we have included the option to select from the 16 included thresholding algorithms in ImageJ. Furthermore, to detect improper image segmentation, we have included the option to check an image immediately after thresholding. In doing so, the user can choose to terminate the process if an image does not appear properly segmented. Another consideration is that the particle identification relies on the size of the primary fluorophore. If the secondary fluorophores, dTomato and Cy5, encompass a larger area than GFP, then the non-overlapping areas are not quantified. This results in some loss of information about each particle and represents a potential weakness of the macro. However, in maintaining a stricter ROI, the potential overlap of adjacent virus is excluded. In addition, quantifying the maximum intensity of Cy5 fluorescence rather than total intensity minimizes the effect of this weakness. Finally, the macro is capable of quantifying intensity from overlapping signals but is not capable of colocalization analysis. However, various plugins are available for use in ImageJ or FIJI to assess colocalization.

In designing this macro, the primary goal was the analysis of the in situ uncoating assay. However, we have included adjustable settings that allow for use outside of the uncoating assay. For example, the HIV viral core has been shown to associate with cellular proteins involved in microtubule trafficking and nuclear import [53]. The Overlap Intensity macro could be used to assess the amount of a cellular protein associated with the viral core at different time points or under different cellular conditions. We have shown signal detection based on GFP which was identified on a green image channel. In cases where the signal of interest is not green, we have included the option to select green, red, blue, gray, cyan, magenta, and yellow channels for ROI identification. User options are expanded further into selecting which channels to measure overlap intensity. Here, we only measured red and blue channels, but all channel options are available. Lastly, all required settings to operate the macro are included or accessible from the user interface. This option includes shortcuts for adjusting image scale and selecting measurement parameters for intensity and area. The macro is openly available for download and use in ImageJ and FIJI at https://github.com/HulmeLab/Overlap-Intensity-Macro (accessed on 11 August 2021). Therefore, we encourage users to test and apply the Overlap Intensity macro to uses beyond the in situ uncoating assay.

## Figures and Tables

**Table 1:** In situ uncoating data from two independent experiments.

Time of Fix Post-Infection (h)	Count	Average % of CA-Positive Virus	Mean Max Cy5 Intensity	Average % of Fusion
BafA	2028	61.6	46.9	16.3
0	770	79.8	60.5	16.3
1	409	68.8	18.4	33.9
2	114	32.8	8.9	18.1
3	67	24.3	3.5	19.1
4	268	31.1	8.5	27.5

## Data Availability

The data presented in this study are contained within the article.
